# Komplikationen und Erfolgsraten des Vena-subclavia-Katheters in Abhängigkeit der Erfahrung

**DOI:** 10.1007/s00101-020-00888-2

**Published:** 2020-11-24

**Authors:** Johannes Schulz, Axel Scholler, Paul Frank, Dirk Scheinichen, Markus Flentje, Hendrik Eismann, Thomas Palmaers

**Affiliations:** 1 Klinik für Anästhesiologie und Intensivmedizin (OE8050), Medizinische Hochschule Hannover, Carl-Neuberg-Str. 1, 30625 Hannover, Deutschland; 2 Anästhesiologische Klinik, Universitätsklinikum Erlangen, Maximiliansplatz 1, 91054 Erlangen, Deutschland

**Keywords:** Zentraler Venenkatheter, Intensivmedizin, Lernkurve, Pneumothorax, Ausbildung, Central venous catheter, Intensive care, Learning curve, Pneumothorax, Education

## Abstract

**Hintergrund:**

Die Punktion der V. subclavia gehört zu den Standardprozeduren eines/einer Anästhesisten/Anästhesistin. Gefürchtete Komplikationen dieser Prozedur sind der Pneumothorax und die arterielle Fehlpunktion. Zum Erlernen dieser Prozedur ist von einer gewissen Lernkurve auszugehen.

**Ziel der Arbeit:**

In dieser Studie soll der Einfluss der Punktionserfahrung auf die Erfolgsquote und mechanische Komplikationen wie Pneumothorax und arterielle Punktion untersucht werden. Dazu sollen 3 Erfahrungsstufen miteinander verglichen werden: unerfahren: 0 bis 20 Punktionen, mäßig erfahren: 21 bis 50 und erfahren: über 50 Punktionen.

**Material und Methoden:**

Post-hoc-Analyse einer vorab publizierten Nichtunterlegenheitsstudie zur Untersuchung des Einflusses der Beatmung auf die Pneumothoraxrate bei der V.-subclavia-Punktion in Landmarkentechnik. Es wurden 1021 Patienten ausgewertet, die in die vorab publizierte Studie zwischen August 2014 und Oktober 2017 eingeschlossen wurden.

**Ergebnisse:**

Die Gesamtrate an mechanischen Komplikationen ist in der Gruppe der Unerfahrenen im Vergleich zur Gruppe der Erfahrenen signifikant höher (15 % vs. 8,5 %, *p* = 0,023). Ebenso ist die Rate an Punktionsversuchen in der Gruppe der Unerfahrenen (0–20) mit 1,85 ± 1,12 signifikant höher als in der Gruppe der Erfahrenen (1,58 ± 0,99, *p* = 0,004). Im Gegenzug war die Rate einer erfolgreichen Punktion mit 95,1 % in der Gruppe der Erfahrenen signifikant höher als in der Gruppe der Unerfahrenen (89,3 %, *p* = 0,001).

**Schlussfolgerung:**

Für eine erfolgreiche Punktion der V. subclavia in Landmarkentechnik ist eine Lernkurve von mindestens 50 Punktionen nötig, um die Komplikationsrate zu senken und die Erfolgsrate zu steigern.

## Abstract (en)

**Background:**

The infraclavicular puncture of the subclavian vein is a standard procedure for anesthetists. Meanwhile the literature and recommendations are clear and the use of real-time ultrasound guidance is the standard procedure; however, anesthetists will always get into special circumstances were they have to use the landmark technique, so this competence must be preserved. Feared complications of infraclavicular subclavian vein puncture are pneumothorax and arterial puncture. Up to now there is no clear learning curve for the infraclavicular subclavian vein puncture in the landmark technique performed by anesthetists.

**Objective:**

The aim of this study was to examine the influence of the puncture experience on the success rate and mechanical complications, such as pneumothorax and arterial puncture in patients who received an infraclavicular subclavian vein puncture with the landmark technique. Three levels of experience were defined for comparison: inexperienced 0–20 punctures, moderately experienced 21–50 and experienced over 50 punctures.

**Material and methods:**

Post hoc analysis of a previously published noninferiority study to examine the influence of ventilation on the pneumothorax rate in the subclavian vein puncture using the landmark technique. This analysis included 1021 anesthetized patients who were included in the original study between August 2014 and October 2017. Demographic data as well as the number of puncture attempts, puncture success, the overall rate of mechanical complications, pneumothorax rate and arterial puncture rates were calculated.

**Results:**

The overall rate of mechanical complications (pneumothorax + arterial puncture) was significantly higher in the inexperienced group (0–21) compared to the experienced group (>50, 15% vs. 8.5%, respectively, *p* = 0.023). This resulted in an odds ratio of 0.52 (confidence interval, CI: 0.32–0.85, *p* = 0.027). Likewise, the rate of puncture attempts in the group of inexperienced (0–20) with 1.85 ± 1.12 was significantly higher than in the group of experienced (>50, 1.58 ± 0.99, *p* = 0.004) and resulted in an odds ratio of 0.59 (CI: 0.31–0.96, *p* = 0.028). Although the puncture attempts of the moderately experienced (21–50) compared to the inexperienced (0–20) was not significant lower, we found an odds ratio of 0.69 (CI: 0.48–0.99, *p* = 0.042). The rate of successful puncture was 95.1% in the experienced group versus 89.3% in the inexperienced group (*p* = 0.001), which resulted in an odds ratio of 2.35 (CI: 1.28–4.31, *p* = 0.018). When viewed individually, no significant differences were found for pneumothorax and arterial puncture.

**Conclusion:**

In this post hoc analysis of the puncture of the subclavian vein using the landmark technique, we found a significant reduction of puncture attempts and overall mechanical complications. At least 50 punctures seem to be necessary to achieve the end of the learning curve; however, the landmark technique should only be used under special circumstances, when real-time ultrasound is not available. Anesthetists who want to complete their repertoire and learn the landmark technique should always perform a static ultrasound examination before starting the puncture in order to reduce complications due to anatomical variations or thrombosis.

## Hintergrund und Fragestellung

Die Anlage eines zentralen Venenkatheters via V. subclavia (VSK) ist eine Standardprozedur in der Anästhesie und Intensivmedizin. Im Vergleich zu anderen Punktionsstellen, wie der V. jugularis interna oder der V. femoralis, verfügt der VSK über eine geringere Thrombose- und Infektionsrate [19, 20, 27]. Auch bei der arteriellen Fehlpunktion ist der VSK überlegen [3, 4, 19, 26]. Dagegen sind der sehr seltene Hämatothorax oder der in 0,1–3,1 % der Fälle vorkommende Pneumothorax häufiger beim VSK zu sehen [3, 8, 12, 18].

Es gibt eine Vielzahl an Studien, die eine ultraschallgestützte Anlage eines zentralen Venenkatheters (ZVK) fordern [11, 15, 21, 28]. Allerdings ist die Datenlage für den VSK noch nicht so eindeutig wie für den Zugang über die V. jugularis interna. In einem Cochrane-Vergleich aus dem Jahr 2015 [5] war bei der landmarkengestützten Punktion für einen VSK lediglich die Rate an arteriellen Punktionen signifikant schlechter als in der ultraschallgestützten Gruppe. Für andere Komplikationen oder gar Erfolgsquoten sowie die Zeitdauer der Anlage konnten dagegen keine Unterschiede gesehen werden. Die genannten Studien fordern aber gleichzeitig, dass die Fertigkeiten zur landmarkengestützten Anlage eines ZVK erhalten bleiben müssen.

In einer angloamerikanischen Umfrage unter Krankenhausärzten [30] zeigte sich, dass trotz der Empfehlungen, die ultraschallgestützte Anlage eines ZVK zu favorisieren, dies noch nicht vollkommen umgesetzt ist. Für die Anlage eines ZVK via V. jugularis interna verwendeten 80 % der Befragten die ultraschallgesteuerte Methode. Bei der V. femoralis waren es nur 45 % und bei der V. subclavia nur 39 %. Als Gründe wurden v. a. die fehlende Verfügbarkeit, eine verlängerte Prozedurenzeit und der Verlust an Fertigkeiten für die landmarkengestützte Variante genannt. Die ultraschallgestützte Punktion in „In-plane“-Technik ist anspruchsvoll und hat eine gewisse Lernkurve [10, 24]. Aber auch die landmarkengestützte Anlage des VSK hat mit Sicherheit eine Lernkurve. So konnten Maizel et al. [17] zeigen, dass junge Assistenzärzte, die die ultraschallgestützte Anlage eines ZVK erlernt hatten, eine hohe Misserfolgsquote und Komplikationsrate hatten, wenn sie aufgefordert wurden, eine landmarkengestützte Punktion durchzuführen. Aktuell gibt es in der verfügbaren Literatur keine ausreichenden Informationen, welchen Einfluss die Erfahrung eines/einer punktierenden Anästhesisten/Anästhesistin bei der landmarkengestützten Anlage eines VSK auf die Komplikationsraten und Erfolgsraten hat. Aus diesem Grund soll anhand einer Post-hoc-Analyse der Daten einer großen prospektiven Studie [25] der Einfluss der Punktionserfahrung bei der Anlage des VSK in Landmarkentechnik auf den Punktionserfolg und die Komplikationen ermittelt werden.

## Studiendesign und Untersuchungsmethoden

Das Studiendesign und die Methode zur Randomisierung wurden bereits vorab veröffentlicht und werden hier erneut kurz dargestellt [25]:

Alle beschriebenen Untersuchungen am Menschen wurden mit Zustimmung der zuständigen Ethik-Kommission (Medizinische Hochschule Hannover 6624-2014, April 2014), im Einklang mit nationalem Recht sowie gemäß der Deklaration von Helsinki von 1975 (in der aktuellen, überarbeiteten Fassung) durchgeführt. Von allen beteiligten Patienten liegt eine schriftliche Einverständniserklärung vor, die vor Einschluss in die Studie und nach ausführlicher Aufklärung eingeholt wurde. Im Zeitraum von August 2014 und Oktober 2017 wurden insgesamt 1250 einschließbare Patienten in 2 deutschen großen Universitätskliniken identifiziert, von denen 1021 Patienten eingeschlossen wurden. Alle Patienten erhielten einen VSK, welcher in beiden Kliniken das Standardvorgehen für ZVK bei elektiven Kraniotomien ist.

Einschlusskriterien waren ein Alter von mindestens 18 Jahren sowie eine elektive Kraniotomie. Ausschlusskriterien waren anatomische Varianten an der Punktionsstelle, Zustand nach Klavikulafraktur, Infektionen an der Punktionsstelle und Schwangerschaft. In der ursprünglichen Fragestellung der Studienpopulation sollte untersucht werden, ob ein Unterschied in der Pneumothoraxrate vorliegt, abhängig davon, ob die Punktion unter Apnoe oder fortgeführter Beatmung durchgeführt wird.

Nach der Randomisierung in die Interventionsgruppe Apnoe oder Beatmung begann die Einleitung einer Allgemeinanästhesie, und es erfolgte eine endotracheale Intubation. Die landmarkengestützte Punktion der V. subclavia wurde in normaler Rückenlage ohne Trendelenburg-Lagerung zur Vermeidung von Hirndruck durchgeführt. Beide Arme wurden angelagert und der Kopf in Neutralstellung positioniert. Auf Lagerungsmaterialien unter der Schulter oder Wirbelsäule wurde verzichtet. Die Punktion erfolgte, außer bei Vorliegen von Kontraindikationen (ipsilateraler Portkatheter- oder Shunt-Verlauf, Axilladissektion etc.), in der Regel auf der rechten Seite.

Das Beatmungsgerät wurde zur Punktion entweder auf manuell/spontan (APL-Ventil 0 mbar) oder volumenkontrollierten Autoflow mit einem Tidalvolumen von 7 ml/kg idealem Körpergewicht (Körpergröße in Zentimetern minus 100) und einem positiven endexspiratorischen Druck (PEEP) von idealem Körpergewicht, geteilt durch 10 in mbar, eingestellt. Die Atemfrequenz wurde entsprechend angepasst, um ein endexspiratorisches CO_2_ von 35–40 mm Hg zu erreichen. Die Katheteranlagen wurden von anästhesiologischen Weiterbildungsassistent*innen mit mindestens einem Jahr Berufserfahrung oder Fachärzten/Fachärztinnen für Anästhesiologie durchgeführt. Die Punktion wurde grundsätzlich unter sterilen Bedingungen gemäß den RKI Richtlinien und in Seldinger-Technik [29] durchgeführt. Zur Anwendung kam ein 30 cm langer, 3‑lumiger ZVK (Fa. Arrow International, Reading, PA, USA) mit 7,5 F. Es wurde zu keinem Zeitpunkt eine Ultraschallunterstützung durchgeführt. Die Punktionstechnik war standardisiert. Die Punktionsstelle wurde ca. 1 cm infraklavikulär am Übergang des medialen zum lateralen Drittel der Klavikula aufgesucht und in Richtung Klavikula vorgeschoben, bis Knochenkontakt spürbar war. Anschließend wurde die Nadel zum Jugulum ausgerichtet, und es wurde unter Aspiration vorgeschoben. Bei Erfolglosigkeit wurde die Nadel bis auf das Hautniveau zurückgezogen, und ein neuer Punktionsversuch mit kranialerer Stichrichtung wurde unternommen. War auch dieser nicht erfolgreich, wurde die Nadel erneut auf Hautniveau zurückgezogen und kaudaler als bei dem ersten Versuch ausgerichtet. Waren alle 3 Punktionsversuche erfolglos, musste die Punktion an eine/n zweite/n Anästhesisten/Anästhesistin mit fachärztlicher Qualifikation übergeben werden. Dieser durfte ebenfalls maximal 3 Punktionsversuche unternehmen, nach dem oben beschriebenen Verfahren. Nach maximal 6 erfolglosen Versuchen wurde die VSK-Anlage abgebrochen, und die Punktion galt als erfolglos. Die Gesamtzahl der Punktionsversuche wurde dokumentiert. Unerfahrene Weiterbildungsassistent*innen wurden bei ihren ersten 20 Punktionen durch einen erfahrenen Fach- bzw. Oberarzt supervidiert, ohne das manuell eingegriffen wurde.

Unabhängig von der Berufserfahrung wurden dabei alle Punktierenden in 3 Kategorien bezüglich der Punktionserfahrung für den VSK eingeteilt. In den Klammern sind jeweils der Erstpunktierende (eine bis max. 3 Punktionen) und der ggf. übernehmende Zweitpunktierende (eine bis max. 3 Punktionen) angegeben. Die übernehmenden Fachärzte verfügten immer über eine Punktionserfahrung >50 VSK:

- Gruppe 0–20 (unerfahren mit Supervision/Facharzt),
- Gruppe 21–50 (mäßig erfahren, keine Supervision/Facharzt),
- Gruppe >50 (Facharzt ohne Supervision/Facharzt).

Nach erfolgreicher Punktion wurde die korrekte Katheterlage mittels intrakardialem EKG und Aspirationsprobe an allen Lumina verifiziert. Eine arterielle Fehllage wurde durch eine venöse Rücklaufprobe ausgeschlossen.

Postoperativ erhielten die Patienten zum Ausschluss bzw. Nachweis eines Pneumothorax eine Sonographie des Thorax. Dazu wurde in 4 Quadranten mittels Seashore-Zeichen oder Barcode-Zeichen im M‑Mode der Pneumothorax ausgeschlossen bzw. verifiziert [1]. Bei positivem Bar-Code-Zeichen wurde eine Thoraxröntgenaufnahme angefertigt, um die Größe und Interventionspflichtigkeit zu bestimmen.

### Statistische Analyse und Fallzahlplanung

Statistische Analysen wurden mit Stata 15.1 (StataCorp LLC, Texas, USA) und SPSS 22 (IBM Deutschland, Ehingen, Germany) durchgeführt.

Alle Variablen wurden mit dem Shapiro-Wilks-Test auf Normalverteilung getestet. Normal verteilte Daten werden als Mittelwerte mit Standardabweichung und Minimum und Maximum dargestellt. Gruppenunterschiede wurden mittels t‑Test für nichtverbundene Stichproben ausgewertet bzw. mittels einer ANOVA mit folgendem Bonferroni-Post-hoc-Test. Dichotome Variablen sind als Häufigkeiten und prozentuale Anteile dargestellt. Unterschiede wurden ebenfalls mittels ANOVA mit folgendem Bonferroni-Post-hoc-Test ermittelt. Die multivariate logistische Regressionsanalyse wurde angewendet, um die „odds-ratio“ mit zugehörigen 95 %-Konfidenzintervallen für Komplikationsraten (einzeln und insgesamt) sowie den Punktionserfolg, adjustiert für die in 3 Stufen kategorisierte Punktionserfahrung, zu ermitteln. Ein *p*-Wert <0,05 wurde in allen Testverfahren als statistisch signifikant angenommen.

## Ergebnisse

In diese Post-hoc-Analyse wurden 1021 Patienten eingeschlossen. Es traten insgesamt 14 Pneumothoraces auf (1,4 %), von denen 9 mittels Thoraxdrainage entlastet werden mussten. Es trat bzw. traten kein Hämatothorax oder sonstige schwere mechanische Komplikationen auf. Die Gesamtkomplikationsrate der gesamten Population betrug 11,9 %, davon entfielen 5,7 % auf arterielle Punktionen. In 92,5 % der Fälle war die Punktion erfolgreich. Die demografischen Daten der Patienten sind in Tab. 1 dargestellt. Es gab in der Studienpopulation innerhalb der 3 Kategorien für die Punktionserfahrung keinen signifikanten Unterschied bezüglich Alter, Gewicht, Größe, BMI oder Geschlecht. Auch waren die Patienten bezüglich der 3 Erfahrungskategorien in beiden Interventionsgruppen homogen verteilt.

**Table 1:** 

*Patientenalter (Jahre)*	*MW* *± SD*	*95* *%-KI*	*Minimum/Maximum*	*Gruppenunterschiede*
0–20	55,4 ± 14,7	53,9–56,8	18–88	0–20 vs. 21–50; *p* = 1,000 0–20 vs. >50; *p* = 0,845 21–50 vs. >50; *p* = 1,000
21–50	56,3 ± 15,9	54,6–58,1	18–87	
>50	56,6 ± 14,8	54,9–58,3	19–88	
*Patientengewicht (kg)*	*MW* *±* *SD*	*95* *%-KI*	*Minimum/Maximum*	*Gruppenunterschiede*
0–20	78,6 ± 17,3	76,9–80,3	44–158	0–20 vs. 21–50; *p* = 1,000 0–20 vs. >50; *p* = 0,851 21–50 vs. >50; *p* = 1,000
21–50	79,2 ± 18,5	77,7–81,2	38–150	
>50	80,1 ± 18,6	78,0–82,2	39–183	
*Patientengröße (kg)*	*MW* *±* *SD*	*95* *%-KI*	*Minimum/Maximum*	*Gruppenunterschiede*
0–20	170 ± 10	169–171	145–200	0–20 vs. 21–50; *p* = 1,000 0–20 vs. >50; *p* = 0,510 21–50 vs. >50; *p* = 1,000
21–50	171 ± 10	170–172	150–199	
>50	171 ± 10	170–173	150–198	
*Patienten, BMI (kg/m*^*2*^*)*	*MW* *±* *SD*	*95* *%-KI*	*Minimum/Maximum*	*Gruppenunterschiede*
0–20	27,0 ± 5,5	26,5–27,6	17,7–61,7	0–20 vs. 21–50; *p* = 1,000 0–20 vs. >50; *p* = 1,000 21–50 vs. >50 *p* = 1,000
21–50	27,0 ± 5,2	26,3–27,5	14,7–49,5	
>50	27,2 ± 5,9	26,6–27,9	14,3–56,5	
*Berufserfahrung (Jahre)*	*MW* *±* *SD*	*95* *%-KI*	*Minimum/Maximum*	*Gruppenunterschiede*
0–20	2,56 ± 1,33	2,43–2,69	1–11	**0–20 vs. 21–50;** ***p*** **=** **0,002** **0–20 vs. >50;** ***p*** **<** **0,001** **21–50 vs. >50; *p*** **<** **0,001**
21–50	4,09 ± 2,91	3,76–4,41	1–18	
>50	14,0 ± 10,13	12,84–15,13	1–35	
*Geschlecht*	*Männlich; n (%)*	*Weiblich; n (%)*	*–*	*Gruppenunterschiede*
0–20	168 (37,4)	232 (40,6)	–	0–20 vs. 21–50; *p* = 0,909 0–20 vs. >50; *p* = 1,000 21–50 vs. >50; *p* = 1,000
21–50	144 (32,1)	170 (29,7)	–	
>50	137 (30,5)	170 (29,7)	–	
*Punktionsbedingungen*	*Apnoe; n (%)*	*Beatmung; n (%)*	*–*	*Gruppenunterschiede*
0–20	193 (39,7)	207 (38,7)	–	0–20 vs. 21–50; *p* = 1,000 0–20 vs. >50; *p* = 1,000 21–50 vs. >50; *p* = 1,000
21–50	152 (31,3)	162 (30,3)	–	
>50	141 (29,0)	166 (31,0)	–	
*Studienzentrum*	*#1; n (%)*	*#2; n (%)*	*–*	*Gruppenunterschiede*
0–20	209 (46,9)	191 (33,2)	**–**	**0–20 vs. 21–50;** ***p*** **=** **0,001** **0–20 vs. >50;** ***p*** **=** **0,018** **21–50 vs. >50; *p*** **<** **0,001**
21–50	108 (24,2)	206 (35,8)	–	
>50	129 (28,9)	178 (31,0)	–	

Demografische Daten der Patienten, abhängig von der Punktionserfahrung (Anzahl der bisher durchgeführten Subclavia-Anlagen) in den 3 Kategorien. Anzahl und prozentualer Anteil sind als *n* (%) ausgewiesen. MW stellt den Mittelwert dar, SD die Standardabweichung und 95 %-KI das 95 %-Konfidenzintervall der Mittelwerte. Gruppenunterschiede wurden mittels ANOVA mit Bonferroni-Post-hoc-Test ermittelt. *p* < 0,05 statistisch signifikant (fett)

In der Verteilung der Punktionserfahrung sowie der Berufserfahrung zeigte sich für alle 3 Kategorien ein signifikanter Unterschied (Tab. 1): Die Kolleg*innen mit den meisten Punktionen hatten auch mehr Berufserfahrung. Die Komplikationsrate insgesamt war mit 15 % in der Gruppe mit 0–20 VSK signifikant höher als in der Gruppe mit mindestens 50 VSK (8,5 %). Auch beim Punktionserfolg gab es zwischen den Unerfahrenen und Erfahrenen einen signifikanten Unterschied (89,3 % vs. 95,1 %) (Tab. 2). Zwischen den Gruppen >50 VSK vs. 21–50 VSK waren diese Unterschiede nicht mehr signifikant. Beim Vergleich der Komplikationsentitäten arterielle Punktion und Pneumothorax konnten keine signifikanten Unterschiede aufgezeigt werden. Bei der Anzahl der benötigten Punktionsversuche war die Gruppe mit einer Erfahrung von mindestens 50 VSK der Gruppe mit nur 0–20 VSK signifikant überlegen (1,58 ± 0,99 vs. 1,85 ± 1,12, *p* < 0,01) (Tab. 2).

**Table 2:** 

*Komplikationen*	*Ja; n (%)*	*Nein; n (%)*	*Gruppenunterschiede*
0–20	60 (15)	340 (85)	0–20 vs. 21–50; *p* = 0,340 **0–20 vs. >50; *p*** **=** **0,023** 21–50 vs. >50 *p* = 0,940
21–50	35 (11,1)	279 (88,9)	
>50	26 (8,5)	281 (91,5)	
*Punktionserfolg*	*Ja; n (%)*	*Nein; n (%)*	*Gruppenunterschiede*
0–20	357 (89,3)	43 (10,7)	0–20 vs. 21–50; *p* = 0,054 **0–20 vs. >50;** ***p*** **=** **0,001** 21–50 vs. >50; *p* = 1,000
21–50	295 (93,9)	19 (6,1)	
>50	292 (95,1)	15 (4,9)	
*Pneumothorax*	*Ja; n (%)*	*Nein; n (%)*	*Gruppenunterschiede*
0–20	5 (1,3)	395 (98,7)	0–20 vs. 21–50; *p* = 0,416 0–20 vs. >50; *p* = 0,883 21–50 vs. >50; *p* = 0,052
21–50	8 (2,5)	306 (97,5)	
>50	1 (0,3 %)	306 (99,7 %)	
*Arterielle Punktion*	*Ja; n (%)*	*Nein; n (%)*	*Gruppenunterschiede*
0–20	30 (7,5)	370 (92,5)	0–20 vs. 21–50; *p* = 0,254 0–20 vs. >50; *p* = 0,283 21–50 vs. >50; *p* = 1,000
21–50	14 (4,5)	300 (95,5)	
>50	14 (4,6)	293 (95,4)	
*Punktionsversuche*	*MW* *±* *SD (Min/Max)*	*95* *%-KI*	*Gruppenunterschiede*
0–20	1,85 ± 1,12 (1–6)	1,73–1,93	0–20 vs. 21–50; *p* = 0,203 **0–20 vs. >50; *p*** **=** **0,004** 21–50 vs. >50; *p* = 0,526
21–50	1,69 ± 1,11 (1–6)	1,57–1,82	
>50	1,58 ± 0,99 (1–6)	1,47–1,69	

Gesamtrate an Komplikationen (Erfolglosigkeit, Pneumothorax und arterielle Fehlpunktion) sowie Einzelbetrachtung der Komplikationen, abhängig von der Punktionserfahrung (Anzahl der bisher durchgeführten Subclavia-Anlagen) in den 3 Kategorien. Anzahl und prozentualer Anteil sind als *n* (%) ausgewiesen. MW stellt den Mittelwert dar, SD die Standardabweichung und 95 %-KI das 95 %-Konfidenzintervall der Mittelwerte. *Min* Minimum, *Max* Maximum. Gruppenunterschiede wurden mittels ANOVA mit Bonferroni-Post-hoc-Test ermittelt. *p* < 0,05 statistisch signifikant (fett)

Dies spiegelt sich in einer 2,35fach höheren Wahrscheinlichkeit (*p* = 0,018) wider, eine Punktion erfolgreich abzuschließen, wenn man im Vergleich zur Gruppe mit unter 20 VSK bereits mehr als 50 VSK-Anlagen durchgeführt hat (Tab. 3). Auch die Odds ratios für Punktionsversuche sind signifikant niedriger für die beiden erfahreneren Gruppen gegenüber 0–20 VSK-Anlagen (OR 0,59 für >50 VSK-Anlagen vs. 0–20 und 0,69 für 21–50 VSK-Anlagen 0–20 (jeweils *p* < 0,05)).

**Table 3:** 

*Komplikationen*	*Odds ratio*	*95* *%-Konfidenzintervall*	*–*
21–50 vs. 0–20	0,71	0,46–1,11	*p* = 0,402
>50 vs. 0–20	0,52	0,32–0,85	***p*** **=** **0,027**
>50 vs. 21–50	0,74	0,43–1,26	*p* = 0,792
*Punktionserfolg*	*Odds ratio*	*95* *%-Konfidenzintervall*	*–*
21–50 vs. 0–20	1,87	1,07–3,28	*p* = 0,087
>50 vs. 0–20	2,35	1,28–4,31	***p*** **=** **0,018**
>50 vs. 21–50	1,25	0,63–2,52	*p* = 1,000
*Punktionsversuche*	*Odds ratio*	*95* *%-Konfidenzintervall*	*–*
21–50 vs. 0–20	**0,69**	0,48–0,99	***p*** **=** **0,042**
>50 vs. 0–20	**0,59**	0,37–0,96	***p*** **=** **0,028**
>50 vs. 21–50	0,86	0,54–1,39	*p* = 1,000
*Arterielle Punktion*	*Odds ratio*	*95* *%-Konfidenzintervall*	*–*
21–50 vs. 0–20	0,58	0,30–1,11	*p* = 0,291
>50 vs. 0–20	0,56	0,31–1,13	*p* = 0,336
>50 vs. 21–50	1,02	0,48–2,19	*p* = 1,000
*Pneumothorax*	*Odds ratio*	*95* *%-Konfidenzintervall*	*–*
21–50 vs. 0–20	2,07	0,67–6,38	*p* = 0,621
>50 vs. 0–20	0,26	0,03–2,22	*p* = 0,654
>50 vs. 21–50	0,13	0,02–1,00	*p* = 0,153

*p* < 0,05 statistisch signifikant (fett)

Chancenverhältnisse (Odds ratio) mit zugehörigen Konfidenzintervallen und Bonferroni-korrigierte Signifikanzniveaus (*p*) für Komplikationen insgesamt sowie für die einzelnen Komplikationen und Erfolgsraten, abhängig von der Punktionserfahrung in den 3 Kategorien

In einer Subgruppenanalyse der Erstpunktierenden (Punktionen 1 bis max. 3) zeigte sich eine signifikante Erhöhung des Punktionserfolges, abhängig von der Punktionserfahrung (Tab. 4).

**Table 4:** 

*Punktionserfolg*	*Ja; n (%)*	*Nein; n (%)*	*Gruppenunterschiede*
0–20	331 (82,8)	69 (17,3)	**0–20 vs. 21–50**; ***p*** **<** **0,001** **0‑20 vs. >50;** ***p*** **<** **0,001** **21–50 vs. >50**; ***p*** **<** **0,001**
21–50	275 (87,6)	39 (12,4)	
>50	278 (90,6)	29 (9,4)	
*Punktionsversuche*	*MW* *±* *SD (Min/Max)*	*95* *%-KI*	*Gruppenunterschiede*
0–20	1,68 ± 0,83 (1–3)	1,60–1,76	0–20 vs. 21–50; *p* = 0,147 **0–20 vs. >50; *p*** **=** **0,003** 21–50 vs. >50; *p* = 0,590
21–50	1,56 ± 0,78 (1–3)	1,47–1,65	
>50	1,48 ± 0,73 (1–3)	1,40–1,56	
*Punktionserfolg*	*Odds ratio*	*95* *%-KI*	*–*
21–50 vs. 0–20	1,47	0,96–2,25	*p* = 0,075
>50 vs. 0–20	2,00	1,26–3,17	***p*** **=** **0,006**
>50 vs. 21–50	1,36	0,82–2,26	*p* = 0,237

Subgruppenanalyse der Erstpunktierenden (maximal 3 Punktionen, bevor der Punkteur gewechselt wurde) bezüglich Erfolglosigkeit und Punktionsversuchen, abhängig von der Punktionserfahrung (Anzahl der bisher durchgeführten Subclavia-Anlagen) in den 3 Kategorien. Anzahl und prozentualer Anteil sind als *n* (%) ausgewiesen. MW stellt den Mittelwert dar, SD die Standardabweichung und 95 %-KI das 95 %-Konfidenzintervall der Mittelwerte. *Min* Minimum, *Max* Maximum. Gruppenunterschiede wurden mittels ANOVA mit Bonferroni-Post-hoc-Test ermittelt. Chancenverhältnisse (Odds ratio) mit zugehörigen Konfidenzintervallen und Bonferroni-korrigierte Signifikanzniveau (*p*) für die Erfolgsraten, abhängig von der Punktionserfahrung in den 3 Kategorien. *p* < 0,05 in allen Tests statistisch signifikant (fett)

## Diskussion

Mit dieser Auswertung konnten aufgezeigt werden, dass die erfolgreiche Anlage eines VSK in Landmarkentechnik maßgeblich von der Punktionserfahrung abhängig ist. Demzufolge haben erfahrene Anästhesieärzte/-ärztinnen mit mind. 50 VSK-Anlagen eine signifikant höhere Erfolgsquote als unerfahrene Ärzte/Ärztinnen (bis zu 20 VSK). Gleichzeitig fallen die Komplikationsraten und die Anzahl der Punktionsversuche von erfahrenen gegenüber unerfahrenen Ärzten/Ärztinnen signifikant geringer aus. Diese Ergebnisse stehen im Einklang mit Daten von Sznajder et al., die ebenfalls nachweisen konnten, dass sich der Punktionserfolg erst nach über 50 durchgeführten Prozeduren signifikant verbessert [31]. Mansfield et al. konnten zeigen, dass steigende Erfahrung in Berufsjahren bei der Anlage eines VSK zu einer signifikanten Verbesserung der Erfolgsquote führt [18]. Im Kontrast zu den genannten Studien konnten Lefrant et al. keinen Zusammenhang zwischen Erfahrung und dem Punktionserfolg oder der Komplikationsrate sehen [16]. Dies steht im Gegensatz zu diesen Ergebnissen, die eine signifikant niedrigere Gesamtkomplikationsrate bei einer Erfahrung von mindestens 50 Punktionen aufzeigt. Der positive Einfluss der Berufserfahrung auf den Punktionserfolg und die Komplikationsrate konnte auch von anderen Autoren aufgezeigt werden [7, 9, 22]. In diesen Studien wurde die Berufserfahrung über den Ausbildungsstand der punktierenden Ärzte/Ärztinnen definiert. Auch in dieser Untersuchung zeigte sich im Vergleich der beiden Studienzentren die Relevanz des Ausbildungsstandes. Im Zentrum #1 war die Gruppe der unerfahrenen Ärzte/Ärztinnen im Vergleich zum Zentrum #2 deutlich größer (33,2 % vs. 46,9 %); dies zeigte sich in den vorab publizierten Ergebnissen entsprechend in den Komplikationsraten der Studienzentren [25].

Die Gesamtrate an Pneumothoraces in den 3 Kategorien (0–20: 1,3 %, 21–50: 2,5 %, >50: 0,3 %; gesamte Population 2,2 %) liegt im üblichen Bereich, der auch von anderen Autoren berichtet wurde [6, 19].

Mit steigender Zahl an Punktionsversuchen treten nachweislich auch mehr Komplikationen auf [13, 16, 18]. Daher wird gefordert, dass die Anzahl der Punktionen auf maximal 3 beschränkt werden soll und danach ein Verfahrenswechsel oder Austausch des Punktierenden stattfinden soll [18, 32]. In dieser Studie lag die Anzahl der Punktionen in allen 3 Gruppen zwischen 1,6 und 1,9 im Mittel. Auch die Gesamtkomplikationsraten fallen relativ niedrig aus. Erklärungen hierfür können die vorgegebene und standardisierte Punktionstechnik sowie die Auswahl der Patienten sein. Die Patienten verfügten über einen grenzwertig normalen BMI, hatten keine Einschränkung der Blutgerinnung und verfügten über eine Normovolämie. Insgesamt also optimale Bedingungen, die die Interpretation unserer Ergebnisse einschränkt. Alle Punktionen wurden im elektiven Tagesprogramm durchgeführt und weisen dadurch per se eine niedrigere Komplikationsrate auf [20]. Des Weiteren wurden die unerfahrenen Punkteure grundsätzlich durch erfahrenere Ärzte/Ärztinnen supervidiert, wodurch die z. T. geringeren Komplikationsraten in der unerfahrensten Gruppe gegenüber der Gruppe mit 21 bis 50 Punktionen zu erklären wäre.

Die häufigste Komplikation bei der Anlage des VSK in unserer Studie war die Punktion der A. subclavia. Im gesamten Kollektiv trat die arterielle Fehlpunktion bei 5,7 % der Patienten auf und entspricht den Ergebnissen von anderen Autoren [7, 19, 20]. Einen signifikanten Unterschied innerhalb der Erfahrungsgruppen (0–20: 7,5 %, 21–50: 4,5 % und >50: 4,6 %) konnte allerdings nicht nachgewiesen werden.

Die im Studienprotokoll vorab festgelegten Erfahrungsgruppen lassen zwar den Schluss zu, dass es mit der Punktionserfahrung eine signifikante Lernkurve bezüglich Punktionserfolg, Zahl der Punktionsversuche und die Gesamtkomplikationsrate gibt, nicht aber in den Einzelkomplikationen Pneumothorax und arterielle Fehlpunktion. Es kann somit nicht ausgeschlossen werden, dass durch die Wahl anderer Erfahrungsgruppen in z. B. 10er-Schritten wie von Merrer et al. [20] ein präziserer und ggf. früherer „cut off“ für einzelne Endpunkte hätte gefunden werden können. Betrachtet man jedoch die Subgruppe der Erstpunktierenden bezüglich des Punktionserfolges alleine (wenn nach 3 Punktionen kein Erfolg erreicht wurde, ist die Punktion als nichterfolgreich gewertet), so zeigt sich dieser Lernkurveneffekt noch deutlicher ab. Trotz Supervision schneidet die Gruppe 0–20 signifikant schlechter ab als die beiden erfahreneren Gruppen. Auch beim Vergleich zwischen den Gruppen 21–50 und mehr als 50 VSK ist die erfahrenere Gruppe signifikant besser, sodass bezüglich des Erfolges eine Lernkurve bis mindestens 50 Punktionen konstatiert werden kann.

Neben den bereits diskutierten gibt es weitere Limitationen in dieser Studie. Die hier untersuchte Anwendung der landmarkengestützten Punktion lässt eine Übertragung unserer Ergebnisse auf die ultraschallgestützte Punktion nicht zu. Die landmarkengestützte Punktion war zum Studienbeginn (2014) in beiden Studienzentren noch der Standard. Durch die mittlerweile ausreichend vorhandene Anzahl an Ultraschallgeräten hat in beiden Kliniken jedoch die Sonographie zur Anlage des VSK als Standard Einzug gefunden. Aufgrund der Ergebnisse zweier Studien [10, 23] empfehlen sowohl die ESA als auch die ASA [2, 14] heute zur Anlage eines VSK zumindest eine sonographische Vorabuntersuchung der Punktionsstelle zur Lokalisation und zur Beurteilung der Gefäße. Idealerweise sollte die Anlage unter Echtzeitsonographie stattfinden.

## Fazit für die Praxis

- In dieser Post-hoc-Auswertung konnte gezeigt werden, dass in einem standardisierten Setting mit Supervision die Gesamtkomplikationsraten einer landmarkengestützten Punktionstechnik sehr gering sein können und eine Lernkurve mit signifikanter Verbesserung nach 50 Punktionen erreicht wird. Die Anzahl an Punktionsversuchen sollte 3 nicht überschreiten, und es muss dann großzügig der Punkteur ausgetauscht oder das Verfahren gewechselt werden.
- Nichtsdestotrotz ist die Echtzeit-Ultraschall-gestützte Punktion im elektiven Umfeld immer die erste Wahl. Gleichsam sollte die landmarkengestützte Punktion erlernt und im Repertoire der Ärzte/Ärztinnen weiter vorhanden sein.
